# HIVconsv Vaccines and Romidepsin in Early-Treated HIV-1-Infected Individuals: Safety, Immunogenicity and Effect on the Viral Reservoir (Study BCN02)

**DOI:** 10.3389/fimmu.2020.00823

**Published:** 2020-05-06

**Authors:** Beatriz Mothe, Miriam Rosás-Umbert, Pep Coll, Christian Manzardo, Maria C. Puertas, Sara Morón-López, Anuska Llano, Cristina Miranda, Samandhy Cedeño, Miriam López, Yovaninna Alarcón-Soto, Guadalupe Gómez Melis, Klaus Langohr, Ana M. Barriocanal, Jessica Toro, Irene Ruiz, Cristina Rovira, Antonio Carrillo, Michael Meulbroek, Alison Crook, Edmund G. Wee, Jose M. Miró, Bonaventura Clotet, Marta Valle, Javier Martinez-Picado, Tomáš Hanke, Christian Brander, José Moltó, The BCN02 Study Investigators

**Affiliations:** ^1^IrsiCaixa AIDS Research Institute-HIVACAT, Badalona, Spain; ^2^Fundació Lluita contra la Sida, Infectious Diseases Department, Hospital Universitari Germans Trias i Pujol, Badalona, Spain; ^3^Faculty of Medicine, Universitat de Vic-Central de Catalunya (UVic-UCC), Vic, Spain; ^4^Department of Cellular Biology, Physiology and Immunology, Universitat Autonoma de Barcelona (UAB), Barcelona, Spain; ^5^Hospital Clinic- IDIBAPS, University of Barcelona, Barcelona, Spain; ^6^Departament d'Estadística i Investigació Operativa, Universitat Politècnica de Catalunya/BARCELONATECH, Barcelona, Spain; ^7^Department of Infectious Diseases, Germans Trias i Pujol Research Institute, Badalona, Spain; ^8^Projecte dels NOMS-Hispanosida, BCN Checkpoint, Barcelona, Spain; ^9^The Jenner Institute, University of Oxford, Oxford, United Kingdom; ^10^Pharmacokinetic/Pharmacodynamic Modeling and Simultation, Institut de Recerca de l'Hospital de la Santa Creu i Sant Pau-IIB Sant Pau, Barcelona, Spain; ^11^ICREA, Barcelona, Spain; ^12^Joint Research Center for Human Retrovirus Infection, Kumamoto University, Kumamoto, Japan

**Keywords:** romidepsin, HDAC inhibitor, kick&kill strategy, HIVconsv, early-treatment

## Abstract

Kick&kill strategies combining drugs aiming to reactivate the viral reservoir with therapeutic vaccines to induce effective cytotoxic immune responses hold potential to achieve a functional cure for HIV-1 infection. Here, we report on an open-label, single-arm, phase I clinical trial, enrolling 15 early-treated HIV-1-infected individuals, testing the combination of the histone deacetylase inhibitor romidepsin as a latency-reversing agent and the MVA.HIVconsv vaccine. Romidepsin treatment resulted in increased histone acetylation, cell-associated HIV-1 RNA, and T-cell activation, which were associated with a marginally significant reduction of the viral reservoir. Vaccinations boosted robust and broad HIVconsv-specific T cells, which were strongly refocused toward conserved regions of the HIV-1 proteome. During a monitored ART interruption phase using plasma viral load over 2,000 copies/ml as a criterium for ART resumption, 23% of individuals showed sustained suppression of viremia up to 32 weeks without evidence for reseeding the viral reservoir. Results from this pilot study show that the combined kick&kill intervention was safe and suggest a role for this strategy in achieving an immune-driven durable viremic control.

## Introduction

Current antiretroviral therapy (ART) effectively suppresses HIV-1 replication, thus preventing disease progression. However, the infection remains chronic given that a latent HIV-1 reservoir, established early after infection, persists despite suppressive ART (1). Upon ART discontinuation, integrated replication-competent proviruses in the reservoir drive a rapid viral rebound (2). Therapeutic vaccination has been proposed as a possible approach to induce an effective immune control able to contain rebounding virus (3).

Most therapeutic vaccines tested in the past expressed one or several HIV-1 proteins, which expanded HIV-1-specific CD8^+^ cytotoxic T lymphocyte (CTL) responses to varying levels. However, the responses were ineffective in controlling viremia after ART interruption, likely because of their suboptimal magnitude, breadth, width, specificity, and/or polyfunctionality (4–8), raising the need for novel immunogens and delivery methods to tackle HIV-1 diversity and the virus' ability to escape. New vaccines strategies are being developed to maximize the vaccine coverage of circulating viruses using multivalent mosaic immunogens designed *in silico* (9, 10). Alternatively, vaccines designs are tested that aim to focus the CTL responses toward more conserved and protective regions of the virus, which are less likely to mutate and escape the T-cell response (11–15). Among the latter, the HIVconsv immunogen is one of the most advanced vaccine candidates in clinical development. HIVconsv immunogen consists of a chimeric protein assembled from 14 highly conserved domains derived from HIV-1 genes Gag, Pol, Vif, and Env alternating, for each domain, the consensus sequence of the four major HIV-1 clades A, B, C, and D (12). Upon delivery to both HIV-1-negative and positive individuals by heterologous prime/boost regimens as DNA or in simian adenovirus of chimpanzee (ChAdV) and poxvirus MVA vectors, HIVconsv vaccines were safe and induced CD8^+^ T cells with broad inhibitory capacity of HIV-1 *in vitro*, but showed no effect on the viral reservoir (16–23).

To overcome the limitations of therapeutic vaccines -administered alone- in targeting the viral reservoir, vaccines are combined with latency reversing agents (LRA) in the so called kick&kill strategies (24). This approach intends to activate transcription of HIV-1 using small molecules able to disrupt the viral latency and facilitate effective sensing and clearance of infected cells by vaccine-elicited HIV-1-specific CTL (25, 26). Histone deacetylase inhibitors (HDACi) have been proposed as potential HIV-1 LRA (27–31). Romidepsin (RMD; Istodax^®^, Celgene Ltd.) is a potent HDACi approved for the treatment of cutaneous and peripheral T-cell lymphomas, which has been shown to induce HIV-1 transcription both *in vitro* and *in vivo* (32, 33). The REDUC trial combined RMD with Vacc-4x and rhuGM-CSF in chronically suppressed HIV-1-positive individuals, resulting in a mean reduction of 39.7% in total HIV-1 DNA (34). However, this intervention failed to delay viral rebound after ART interruption, suggesting that the reservoir-purge effect was not sufficient and/or the vaccine-induced response was unable to eliminate cells actively replicating HIV-1. In fact, the increase in cell-associated HIV-1 RNA inversely correlated with time to rebound, supporting that, in the absence of an enhanced HIV-1-specific CTL response, viral reactivation might facilitate viral rebound once ART is interrupted (35).

Here, in this single-arm, open-label, phase I, proof-of-concept study, referred to as BCN02 trial (NCT02616874), we assessed the safety, tolerability, immunogenicity and effect on the viral reservoir of a kick&kill strategy consisting of the combination of HIVconsv vaccines with RMD in suppressed early-treated HIV-1-infected individuals. Participants were rolled-over from the therapeutic vaccine trial BCN01 (NCT01712425), in which individuals who started ART during acute/recent HIV-1 infection had received a prime/boost regimen of the ChAdV63.HIVconsv and MVA.HIVconsv vaccines (CM) (20). Three years after, BCN01 participants who had shown sustained viral suppression and who accepted to participate in BCN02 study were immunized with two doses of MVA.HIVconsv, before and after three weekly-doses of RMD, followed by a monitored antiretroviral pause (MAP) for a period of 32 weeks to assess the ability of the intervention to control viral rebound.

## Materials and Methods

### Study Design and Interventions

The BCN02 clinical trial was an investigator initiated phase I, open-label, single-arm, multicenter, single-country study to assess the safety, tolerability and efficacy of a combined kick&kill strategy in suppressed HIV-1-infected patients that had initiated ART during acute/recent HIV-infection. Individuals were rolled over from vaccine trial BCN01 (20) and invited to participate after 3 years on suppressive ART. A complete list of inclusion/exclusion criteria is available in the Study Protocol (Appendix). The study took place between February 2016 and October 2017 at two HIV-1 units from universitary hospitals (Hospital Universitari Germans Trias i Pujol -HUGTIP, Badalona and Hospital Clínic, Barcelona) and a community center (BCN-Checkpoint, Barcelona). Before inclusion in the study, all participants signed an informed consent previously discussed, reviewed and approved by the Community Advisory Board of the Barcelona-based vaccine program (HIVACAT). The study was approved by the institutional ethical review board of the participating institutions (Reference Nr AC-15-108-R) and by the Spanish Regulatory Authorities; and was conducted in accordance to the principles of the Helsinki Declaration and local personal data protection law (LOPD 15/1999). The MVA.HIVconsv vaccine was GMP manufactured at IDT Biologika GmbH, Germany, and supplied for the study under an investigator initiated clinical trial contract agreement. Risk of Genetically Modified Organism release to the environment was evaluated by the Spanish Ministry of Environment (B/ES/12/09). RMD was supplied for the study by Celgene Ltd. (Couvet, Switzerland) under an investigator initiated clinical trial contract agreement.

**Figure 1:**
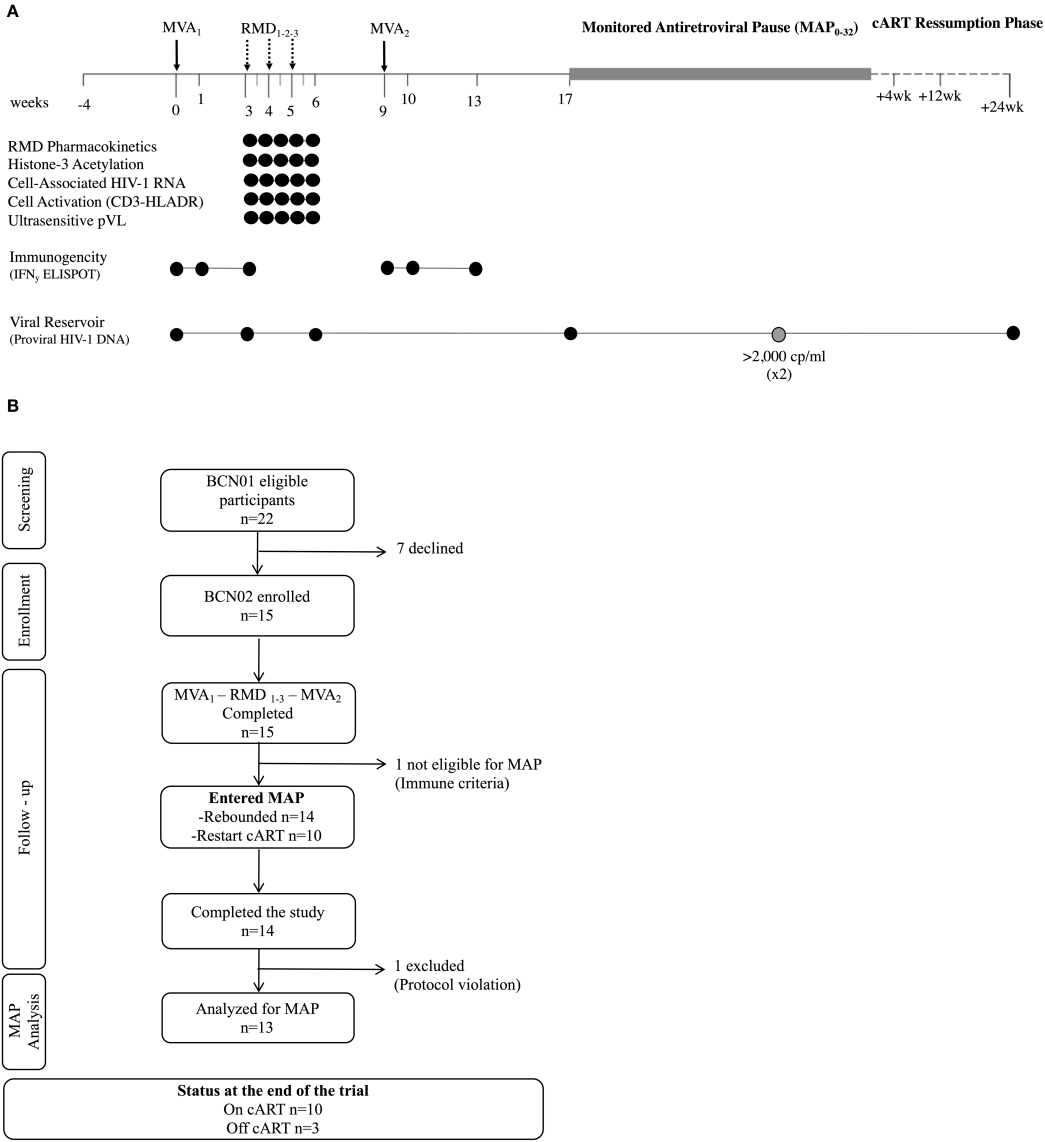
Trial design. **(A)** Schematic study design. **(B)** Consolidated Standards of Reporting Trials (CONSORT) flow diagram for the trial. ^*^Viral rebound during MAP was defined as pVL >20 copies/ml and ^#^criteria for ART resumption included pVL over 2,000 copies/ml in two consecutive determinations, CD4^+^ cell counts decrease over 50% and/or below 500 cells/mm^3^ and/or development of clinical symptoms suggestive of an acute retroviral syndrome. MVA, MVA.HIVconsv vaccine; RMD, romidepsin; MAP, monitored antiretroviral pause; ART, antiretroviral therapy; pVL, plasma HIV-1 viral load.

The BCN02 trial design is summarized in Figure 1A. After their inclusion in the study (week 0), all participants received a first dose of 2 × 10^8^ plaque-forming units (pfu) of MVA.HIVconsv (MVA_1_) administered intramuscularly, followed by three weekly doses of RMD of 5 mg/m^2^ BSA infused intravenously over 4 hours (RMD_1−2−3_), and by a second dose of 2 × 10^8^ pfu of MVA.HIVconsv (MVA_2_) 4 weeks after RMD_3_ to compensate for any potential impairment in the previous vaccine-induced response caused by RMD. Following RMD prescription information, participants received prophylactic antiemetic treatment with ondasetron before and during 3 days after each RMD dose.

Eight weeks after MVA_2_ (week 17), eligible participants initiated a MAP for a maximum of 32 weeks (MAP_0−32_) or until any ART resumption criteria were met. To be eligible for the MAP, participants had to maintain undetectable HIV-1 pVL and meet the immune futility criteria, defined as showing a net increase in HIVconsv-specific immune response with MVA_2_ boost measured in an *ex vivo* IFN-γ ELISPOT assay. During MAP, participants were allowed to choose either the hospital units or BCN-Checkpoint community center for the follow up visits. Symptoms suggestive of acute retroviral syndrome and sexually transmitted diseases were solicited and viral load was tested using the finger-tip Xpert HIV-1 Qual kit (Cepheid, Sunnyvale, CA, US) in all visits. When a positive result was obtained in the Xpert HIV-1 Qual, participants were called in for a confirmatory quantitative pVL within the next 24 h. If pVL was confirmed to be over 20 copies/ml, a visit was scheduled 3 days after to closely monitor viral rebound and be able to offer prompt ART resumption if required. Details on viral load management during MAP are described in the Study Protocol (Appendix). After ART ressumption, participants were followed at 4, 12, and 24 weeks to assure that they re-attained viral suppression.

### Study Population

BCN02 participants were adult (≥18 years) HIV-1-infected individuals, who had initiated ART >6 months after estimated date of HIV-1 acquisition and who had received a prime/boost heterologous vaccination regimen using ChAdV63.HIVconsv-MVA.HIVconsv in the parental BCN01 study (20). To be eligible for BCN02, participants had to maintain optimal HIV-1 suppression during at least 3 years and CD4^+^ cell counts ≥500 cells/mm^3^ at BCN02 baseline visit. Main exclusion criteria included active hepatitis B or C, history of AIDS-defining disease, treatment for cancer or lymphoproliferative disease within 1 year before study entry or use of immunosuppressants within the 3 months prior to the screening visit. Concomitant treatment with strong CYP3A4 inhibitors was not permitted, but switching ART to a non-boosted integrase-inhibitor raltegravir- or dolutegravir-based regimen at least 4 weeks before baseline visit was allowed for those patients receiving ART containing ritonavir or cobicistat at screening.

### Study Endpoints

The primary endpoint of this study was to assess the safety, tolerability and the effect on the viral reservoir size of the combined treatment with HIVconsv vaccines and RMD given as a latency reversing agent. Secondary endpoints included the extent and specificity of the CTL response and the effect of the intervention in controlling viral rebound after ART interruption. Other secondary endpoints included RMD pharmacokinetics and the effects of RMD on histone acetylation in lymphocytes, induction of viral transcription, changes in T-cell activation surface markers, and quantification of plasma viremia.

Safety and tolerability were evaluated by the development of grade ≥3 and serious adverse events (AE). Local and systemic AE were solicited prospectively for a minimum of 7 days following each immunization and RMD administration. Both local and systemic AE were graded according to the Division of AIDS (DAIDS) Table for Grading the Severity of Adult and Pediatric Adverse Events, version 2.0, November 2014, accessible online at https://rsc.niaid.nih.gov/sites/default/files/daids-ae-grading-table-v2-nov2014.pdf. AE were specified as unrelated, unlikely, probably or definitely related to the investigational products by the investigator.

### Determination of RMD Pharmacokinetics

The concentration of RMD in plasma was determined, for RMD_1_, at the end of the infusion (4 h) and 4.5, 5, 6, 8, 12, and 24 h after and, for RMD_2_ and RMD_3_, at the end of the infusion and 12 h after. RMD concentrations were measured by liquid chromatography-mass spectrometry/mass spectrometry (LC-MS/MS), according to a validated method. A population pharmacokinetic model for RMD was developed using non-linear mixed-effects modeling with the computer program NONMEM version 7.3 (Icon Development Solution, Ellicot City, MD) (36). Bayesian estimates of the individual parameters of RMD were used to simulate individual drug concentrations, and RMD area under the concentration-time curve (AUC0-inf) was calculated for each individual on each occasion using a non-compartmental approach (Winnonlin software; Phoenix, version 7.0).

### Flow Cytometry Determination of acH3 and Activation of T Cells

The levels of histone H3 acetylation in lymphocytes (based on FSC/SCC scatter) were determined by flow cytometry from samples taken before (0 h) and at the end of each RMD infusion (RMD_1−2−3_) (4 h), at 8 and 24 h (+1 day) RMD_1_, and at 72 h (+3 days) and 7 days after (RMD_1−2−3_). Cryopreserverd PBMC were thawed 4 h before use, and 500,000 cells were blocked with 600 ul of PBS/10% FBS for 20 min and stained with polyclonal rabbit anti-acetyl histoneH3 (10 μg/ml, MerckMillipore #06–599) or normal rabbit serum (control stain, LifeTechnologies #10510) for 30 min. Cells were washed and subsequently incubated with donkey anti-rabbit IgG(H+L) (6 μg/mL, LifeTechnologies #A21206) for 30 min at room temperature in the dark. Cells were washed, re-suspended in 150 μl PBS and analyzed. ~50,000 events were acquired per sample. The median fluorescence intensity (MFI) for each sample was calculated by substracting the background MFI (isotype control stain) from the anti-acetyl histoneH3 stain.

Activation of T cells was determined based upon HLA-DR expression on CD3^+^ T cells. Cryopreserved PBMC were thawed, and 1,000,000 cells were stained with CD3 APC-Cy7, CD4 FITC, CD8 BV510 and HLA-DR PECy7 (BioLegend #344818, 300538, 301048, and 307616, respectively). Cells were collected on an LSRII instrument (BD), and data analyzed according to the gating criteria shown in Supplementary Figure 1 using FlowJo 10 software.

### Quantification of Cell-Associated (CA) HIV-1 RNA in CD4^+^ T Cells

Cell-associated HIV-1 RNA was quantified in peripheral CD4^+^ T cells by ddPCR (One-Step RT-ddPCR Advanced Kit for Probes, BioRad) from samples taken before (0 h) and at the end of each RMD infusion (4 h), at 8 and 24 h (+1 day) after RMD_1_, and 72 h (+3 days) and 7 days after RMD_1−2−3_. CA HIV-1 RNA was quantified using two different primers/probe sets annealing to the 5′LTR and GAG conserved regions of HIV-1, to circumvent potential primer mismatch in individuals' viral sequence as previously described (37). HIV-1 transcription levels were normalized to the housekeeping gene TATA-binding protein (TBP) and shown as relative to levels before RMD_1_.

### Ultra-Sensitive Determination of Residual Viremia

To evaluate HIV-1 RNA below 20 copies/ml, 4–8 ml of plasma samples taken before (0 h) and at the end of each RMD infusion (4 h), at 8 and 24 h (+1 day) after RMD_1_, and 72 h (+3 days) and 7 days after RMD_1−2−3_ were ultracentrifugated at 170,000 g at 4°C for 30 min and viral RNA was extracted automatically using the m2000sp Abbot device. HIV-1 RNA copies were quantified using the Abbott Real-Time HIV-1 assay (Abbott Molecular Inc.) and in-house calibration curve sets as described (38). The limit of detection (2 HIV-1 RNA copies/mL) was calculated relative to the plasma volume.

### Vaccine Immunogenicity

Total HIV-1 and HIVconsv-specific T cells were assessed *ex vivo* using cryopreserved PBMC obtained the day of vaccination and 1 week afterwards, 3 weeks after MVA_1_, and 4 weeks after MVA_2_ using an IFN-γ-detecting enzyme-linked immunoabsorbent spot assay (ELISPOT IFN-γ Mabtech kit). 15-mer peptides overlapping by 11 amino acid were combined into 6 pools of 32–33 peptides per pool corresponding to the HIVconsv vaccine insert (P1-P6, total *n* = 166 peptides, IN pools) (**Figure 3A**) and 12 pools of 39–67 peptides per pool spanning the rest of the HIV-1 viral protein sequences (OUT pools for “outside the immunogen,” obtained through the NIH AIDS Reagent Program). All peptides pools were tested in duplicates. The final concentration of individual peptides in the ELISPOT assay was 1.57 μg/ml. Medium only was used as no-peptide negative control in quadruplicate wells, and PHA (50 μg/ml) and a CEF peptide pool (2 μg/ml) consisting of 23 previously defined human CD8^+^ T-cell epitopes from cytomegalovirus, Epstein-Barr virus and influenza virus (C.T.L. OH, USA) were added as positive controls.

To address the breadth of the vaccine-induced response at the peak immunogenicity time point, an IFN-γ ELISPOT assay with *in vitro* expanded T cells was performed on stored samples from week 10 and 13 to test individual overlapping peptides covering the HIVconsv immunogen sequence (*n* = 166 OLP). Briefly, cryopreserved PBMC were thawed and incubated for 3 h at 37°C in R10 before stimulation with an anti-CD3 monoclonal antibody during 2–4 weeks in RPMI 1640 supplemented with FBS and penicillin/streptomycin with 50 U/ml of recombinant IL-2 (39). Before their use in ELISPOT assays, the expanded cells were washed twice with R10 and incubated overnight at 37 °C in the absence of IL-2.

Spots were counted using an automated Cellular Technology Limited (C.T.L., OH, USA) ELISPOT Reader Unit. The threshold for positive responses was set at ≥50 SFC/10^6^ PBMC (5 spots per well), > the mean number of SFC in negative control wells plus 3 SD of the negative control wells, or > 3 × the mean of negative control wells, whichever was higher. To avoid overestimating the breadth of responses, positive responses to two consecutive 15-mer overlapping peptide were counted as one response. The highest magnitude of the sequential responses was taken as the magnitude for each identified response.

### Quantification of HIV-1 Reservoir

To quantify the size of the peripheral blood proviral reservoir, lysed extracts from CD4^+^ T cells were used to measure total CA HIV-1 DNA by ddPCR. Primers and probes for the RPP30 cellular gene were used for input normalization.

### Statistical Analysis

Qualitative variables were represented as mean absolute and relative frequencies, whereas quantitative variables were represented as mean or median and range. Safety endpoints are summarized by the number and percentage of participants reporting local and systemic AE and their grading. The Wilcoxon signed rank test was used to test whether the viral reservoir and the immune parameter changed as an effect of the intervention, without correction for multiple comparisons. The maximum breadth of the T-cell response per individual was estimated as the number of P1-P6 pools eliciting a positive response throughout the study and the number of individual OLP eliciting a response at peak immunogenicity time point from the mapping assay. Reservoir size and immunogenicity were analyzed using GraphPad Prism (v5.01) for Mac OS X (San Diego, CA).

To evaluate the effect of the intervention on viral rebound, a positive pre-defined efficacy signal was established if at least over 20% of patients remained with pVL below 2,000 copies/ml at week 12 of MAP, considering previous data suggesting that early treatment initiation could favor delayed viral rebound/spontaneous viral control in up to 15% of individuals (40). However, BCN02 was an exploratory pilot trial and, due to the absence of a control arm and its final small sample size, the nature of this study only allowed to detect trends in virological effects, which collectively, could be useful to design future studies. To detect possible factors associated to the viremic control observed during the MAP phase, univariate log-binomial regression models were used (41). This model uses the logarithm as a link function, and is a generalized lineal model for a binary outcome where the error terms follows a binomial distribution. The effect size measure of the model is the relative risk. Because of the low number of MAP-C (*n* = 3), multivariate log-binomial regression models were not fitted. The significance threshold for all univariate analyses was set at a two-sided α = 0.05. The analyses were performed with R Core Team (42) (v3.0.2).

## Results

### Participants Enrolled in the Study

Between February 29th and September 15th 2016, 15 out of the 22 eligible BCN01 participants were enrolled in BCN02. Seven declined to participate due to their inability to attend all the scheduled visits. Baseline characteristics of trial participants are summarized in Table 1. All 15 participants received two doses MVA.HIVconsv (MVA_1−2_) and three doses of romidepsin (RMD_1−2−3_) as shown in the study chronogram (Figure 1A), and were included in the safety, immunogenicity and reservoir analyses. One participant was not eligible for MAP due to immune futility pre-defined criteria and 14 participants underwent a MAP for a maximum of 32 weeks. Retrospective analyses of stored plasma samples obtained during MAP revealed the presence of antiretroviral drugs in some samples from one participant, whose MAP data were censored for the viral rebound kinetics analyses (Figure 1B).

**Table 1:** Demographic, clinical, and treatment characteristics of study patients at study entry (*n* = 15).

Age (years)	43 (33–51)
Gender (M/F), *n*	14/1
MSM/HTS, *n*	14/1
Time since HIV-1 acquisition to ART (days)	92 (28–164)
Pre ART log_10_ HIV-1 RNA (copies/ml)	4.9 (3.2–5.8)
Time on ART (years)	3.23 (3.03–3.77)
ART regimen, *n (%)*	
TDF/FTC/RAL	11 (73)
ABC/3TC/RAL	2 (13)
ABC/3TC/DTG	2 (13)
CD4^+^ T-cell counts (cells/mm^3^)	728 (416–1,408)
Ratio CD4/CD8	1.37 (0.97–1.93)

*Median (range) is shown unless otherwise described. M, male; F, female; MSM, men who have sex with men; HTS, heterosexual; ART, antiretroviral therapy; TDF, Tenofovir Disoproxil Fumarate; FTC, Emtricitabine; RAL, Raltegravir; ABC, Abacavir; 3TC, Lamivudine; DTG, Dolutegravir*.

### Safety of MVA.HIVconsv and RMD Administrations

All participants reported adverse events (AE) related to both study investigational medicinal products. A total of 333 AE were recorded during the study intervention phase, 129 after MVA_1−2_ and 204 after RMD_1−2−3_, which were mostly mild or moderate (grade 1-2) (*n* = 318, 95%). The most frequent AE related to MVA.HIVconsv, summarized in Table 2, were local pain at the injection site and a flu-like syndrome consisting of fatigue, headache, myalgia, and/or low-grade temperature (<38°C). Regarding AE related to RMD (Table 3), the most common grade 1–2 events were headache, fatigue, and gastro-intestinal symptoms. Despite prophylactic ondansetron treatment, 4 (27%) individuals vomited the days of RMD administration. One participant experienced a grade 4 AE consisting in a sepsis by *Shigella sonnei* that required hospital admission for 24 h, thus fulfilling the criteria of serious adverse event (SAE). The symptoms started within 4 h after RMD_3_ and therefore, the SAE was considered as possibly related to RMD.

**Table 2:** Summary of adverse events related to MVA.HIVconsv vaccinations (*n* = 15).

	**Grade 1 *n***	**Grade 2 *n***	**Grade 3 *n***	**Grade 4 *n***	**Total *n (%)***
**Injection site reaction**					
Local pain	7	4	2	0	13 (87)
Redness	1	0	0	0	1 (7)
Induration	0	0	0	0	0 (0)
**Systemic adverse events**					
Fatigue	7	4	2	0	13 (87)
Headache	5	3	1	0	9 (60)
Myalgia	4	3	2	0	9 (60)
Fever	5	0	0	0	5 (33)
Anorexia	3	0	1	0	4 (27)
Sweating	2	2	0	0	4 (27)
Nausea	2	0	1	0	3 (20)
Abdominal pain	0	1	1	0	2 (13)
Flatulence	1	0	0	0	1 (7)
Somnolence	1	0	0	0	1 (7)

**Table 3:** Summary of adverse events related to RMD_1−2−3_ treatment (*n* = 15).

	**Grade 1 *n***	**Grade 2 *n***	**Grade 3 *n***	**Grade 4 *n***	**Total *n (%)***
Headache	9	5	0	0	14 (93)
Fatigue	9	5	0	0	14 (93)
Nausea	4	7	0	0	11 (73)
Anorexia	8	1	0	0	9 (60)
Abdominal pain	5	2	0	0	7 (47)
Metallic taste	5	1	0	0	6 (40)
Constipation	4	2	0	0	6 (40)
Abdominal distension	4	1	0	0	5 (33)
Vomits	4	0	0	0	4 (27)
Sweating	2	2	0	0	4 (27)
Palpitations	3	0	0	0	3 (20)
Myalgia	1	1	0	0	2 (13)
Rash	0	2	0	0	2 (13)
Dry mouth	1	0	0	0	1 (7)
ECG: ST-elevation	1	0	0	0	1 (7)
Anxiety	0	1	0	0	1 (7)
Libido decrease	0	1	0	0	1 (7)
Somnolence	1	0	0	0	1 (7)
Sepsis by *Shigella sonnei* (SAE)	0	0	0	1	1 (7)
Hypotension	0	1	0	0	1 (7)

No laboratory abnormalities related to MVA_1−2_ were reported. All laboratory abnormalities related to RMD were grade 1-2 (*n* = 22), the most frequent being hypophosphatemia (8 events) and thrombocytopenia (5 events), except from one case of grade 4 creatinine kinase elevation with normal eGFR which resolved within 7 days without sequelae. Noteworthy, CD4^+^ T-cell counts showed a transient decrease by a median of 248 cells/mm^3^ 3 days after each RMD administration which was not fully recovered by day 7 after RMD_3_ (Supplementary Figure 2). Overall, both MVA.HIVconsv and RMD at the regimen and dose administered in this study were well-tolerated and safety profiles were consistent with data previously reported (20, 34).

During the MAP, 12 (86%) participants reported a total of 58 AE, which were all grade 1-2 and not suggestive of acute retroviral syndrome (not shown). Grade 1 anxiety was observed in one participant who repeatedly declined psychological support (same participant with protocol violation during the MAP).

### RMD Pharmacokinetics and Pharmacodynamics

**Figure 2:**
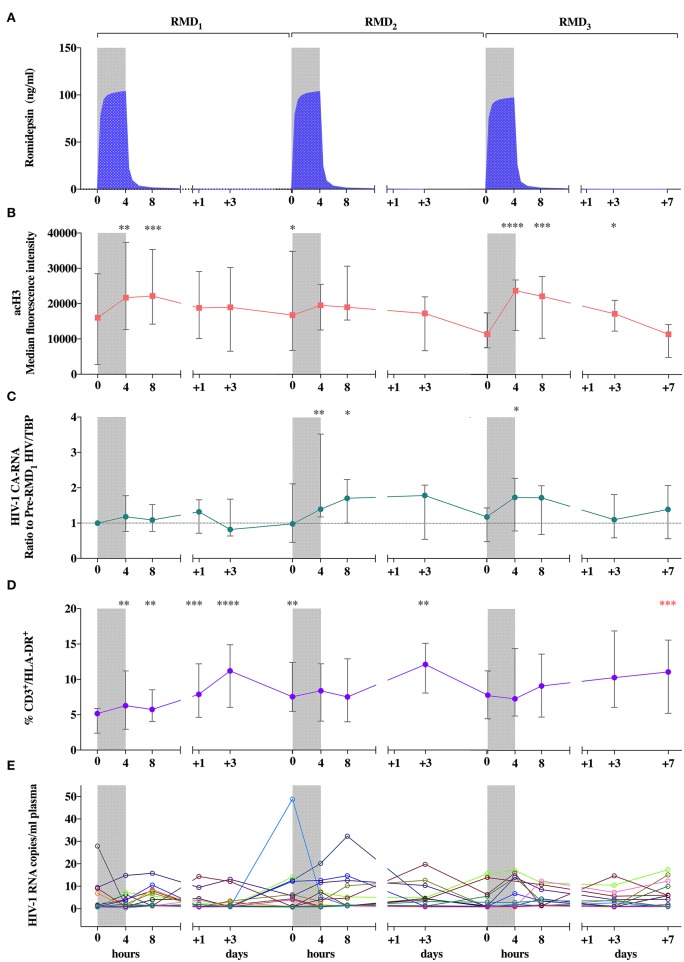
Pharmacokinetic and pharmacodynamic effects of RMD. **(A)** Mean individual predictions of RMD plasma concentrations. **(B)** Levels of histone H3 acetylation in peripheral lymphocytes. **(C)** Viral transcription levels expressed as changes from pre-RMD_1_ levels of cell-associated HIV-1 RNA in peripheral CD4^+^ T-cells. **(D)** Levels of T-cell activation (CD3^+^/HLA-DR^+^ cells). **(E)** individual determinations of pVL. Median of frequencies and IQR (error bars) are represented. Wilcoxon signed-rank *p*-values compare each represented time point with the corresponding values preceding each RMD administration. ^*^p < 0.05. ^**^p < 0.01. ^***^p < 0.001 and ^****^p < 0.0001. The *p* value resulting from the comparison between the value at day 0 of RMD_1_ and 7 days after RMD_3_ is shown in red.

Pharmacokinetics of RMD was comparable to previously described data (43). A population pharmacokinetic model adequately describing RMD concentrations in plasma was developed (36). According to the individual profiles simulated using the model, each infusion was followed by a rapid and polyexponential decline in RMD concentrations in plasma, reaching nearly undetectable levels by 24 h after dosing (Figure 2A).

Regarding the direct effect of RMD on chromatin and induction of viral transcription, histone H3 acetylation (acH3) increased rapidly during each RMD infusion, remained high during 4 h, and returned to baseline values 3 days after each dose (Figure 2B), which is consistent with previous reports (33, 34). HIV-1 transcription transiently increased in parallel, with changes more pronouned after RMD_2_ and RMD_3_ (Figure 2C). These changes were more evident without normalization for house-keeping genes (Supplementary Figure 3) possibly reflecting the general increase in histone acetylation levels (44) induced by RMD. Increases in T-cell activation, measured by the proportion of CD3^+^/HLA-DR^+^ cells, were observed 3 days after each RMD dose. Over the course of the three RMD doses, T-cell activation increased in a progressive manner and was maintained up to 1 week after RMD_3_ (Figure 2D), suggesting a cumulative effect of RMD.

To evaluate changes in levels of quantifiable plasma HIV-1 RNA, an ultrasensitive single copy assay was used. Kinetics of plasma HIV-1 RNA levels did not follow a clear pattern (Figure 2E), despite the increasing percentage of participants with detectable low-level viremia at the end of each RMD dose (Supplementary Figure 4). Collectively, we reproduced effects on acH3, HIV-1 transcription and T cell activation previously reported in chronically infected individuals (34), suggesting that a lower viral reservoir level achieved by early-treatment initiation does not preclude the reactivation potential of RMD.

### MVA.HIVconsv Immunogenicity

**Figure 3:**
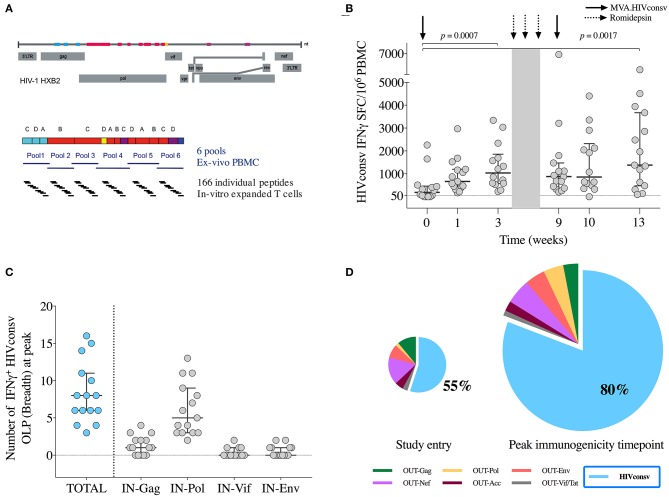
Vaccine immunogenicity. **(A)** Schematic representation of the selected regions in the HIV-1 proteome from different clades included in the HIVconsv immunogen and distribution of 6 peptide pools (P1-P6) and individual overlapping 15-mer peptides (OLP) used in the IFN-γ ELISPOT assays. **(B)** Magnitude (sum of SFU/10^6^ PBMC to pools P1-P6) of vaccine-induced responses over the BCN02 study. Horizontal and error bars represent median and IQR, respectively, and *p*-values correspond to comparisons between the indicated time points using the Wilcoxon signed-rank test. **(C)** Breadth of vaccine-elicited responses toward individual OLP included in the indicated HIVconsv regions. Horizontal and error bars represent median and IQR, respectively. **(D)** Average distribution of total HIV-1 T-cells according to their specificity at the indicated time points. HIVconsv-specific responses are shown in blue. Pie charts are scaled according to the total frequencies of responses. IN are peptide pools corresponding to the HIVconsv vaccine insert and OUT peptide pools spanning the rest of HIV-1 proteome “outside the immunogen”.

Total HIV-1 and HIVconsv-specific T cells were assessed *ex vivo* by an IFN-γ-detecting enzyme-linked immunoabsorbent spot (ELISPOT) assay using 6 peptide pools covering the HIVconsv immunogen sequence (P1-P6) on week 0 (day of MVA_1_), 1, 3 (day of RMD_1_), 9 (day of MVA_2_), 10, and 13. A total of 90 samples were obtained, of which 3 (3%) were censored due to low positive controls and/or high background. All 15 participants showed an absolute increase in HIVconsv-specific IFN-γ-producing T cells during the study, either after MVA_1_ (Wilcoxon signed-rank, *p* = 0.0007) or after MVA_2_ (Wilcoxon signed-rank, *p* = 0.0017) (Figure 3B). Median (range) total frequencies of HIVconsv-specific T cells reached 1,965 (530-6,901) spot-forming cells (SFC)/10^6^ PBMC at the peak immunogenicity time point, which represented an absolute median increase of 1,600 (300–6,621) SFC/10^6^ PBMC from baseline (Wilcoxon signed-rank, *p* < 0.0001).

Over the intervention phase, participants responded to median (range) of 5 (2–6) peptide pools (Supplementary Figure 5). To map the maximum vaccine-induced breadth at peak immunogenicity time point (weeks 10–13), *in vitro* expanded T cells responding toward individual OLPs covering the HIVconsv immunogen were assessed. Median (range) of 8 (3–16) IFN-γ-producing responses to individual OLPs were found, with a dominance in Pol-specific T cells, consistent with the immunogen composition (Figure 3C).

The dominance of HIVconsv-specific responses was calculated at each time point as the percentage of HIVconsv-specific T-cell frequencies divided by the total HIV-1 proteome-specific T-cell frequencies. At the moment of HIV diagnoses, HIVconsv responses were subdominant (<10% being HIVconsv-specfic) and peaked after the CM vaccination reaching a median (range) of 58% (7%−100%) of the total anti-HIV-1 T-cell responses (BCN01 parental study) (20). In BCN02, 2 years from the last HIVconsv vaccination, the increase in the frequency of HIVconsv-specific T-cell responses after MVA_1_ or MVA_2_ further shifted the patterns of T-cell immuno-dominance toward HIVconsv with median (range) of 85% (54%–100%) of the total anti-HIV-1 T-cell responses at peak immunogenicity time point being HIVconsv specific (Figure 3D).

### Effects on the HIV-1 Reservoir

**Figure 4:**
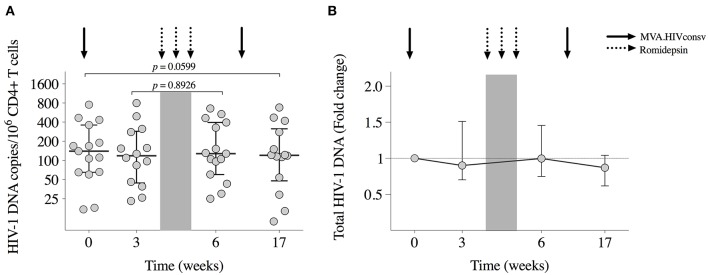
Viral reservoir. **(A)** Total HIV-1 DNA copies/10^6^ CD4^+^ T cells for each participant are shown at study entry (week 0), week 3 (day of RMD_1_), and week 6 (1 week after RMD_3_) and at week 17 (8 weeks after MVA_2_). Horizontal and error bars represent median and IQR, respectively, and *p*-values correspond to comparisons between the indicated time points using the Wilcoxon signed-rank test. **(B)** Changes in proviral DNA throughout the study with respect to baseline (week 0), which is considered to be 1.

All participants had detectable viral reservoirs, as measured by total CD4^+^ T cell-associated HIV-1 DNA, throughout the study. Results from 2 samples out of a total of 60 were considered invalid and were censored. At BCN02 study entry, median (range) reservoir size was of 140 (17–752) HIV-1 DNA copies/10^6^ CD4^+^ T-cells (Figure 4). Proviral DNA showed a tendency to further decrease from baseline to week 17 (Wilcoxon signed-rank, *p* = 0.0599, Figure 4) to median (range) levels of 120 (11-680) copies/10^6^ CD4^+^ Tcells.

### Monitored Antiretroviral Pause (MAP)

Participants undergoing MAP were monitored weekly for the first 12 weeks and every 2 weeks thereafter for a maximum of 32 weeks (MAP_0−32_). Criteria for ART resumption included pVL over 2,000 copies/ml in two consecutive determinations, CD4^+^ cell counts decrease over 50% and/or below 500 cells/mm^3^ and/or development of clinical symptoms suggestive of an acute retroviral syndrome.

**Figure 5:**
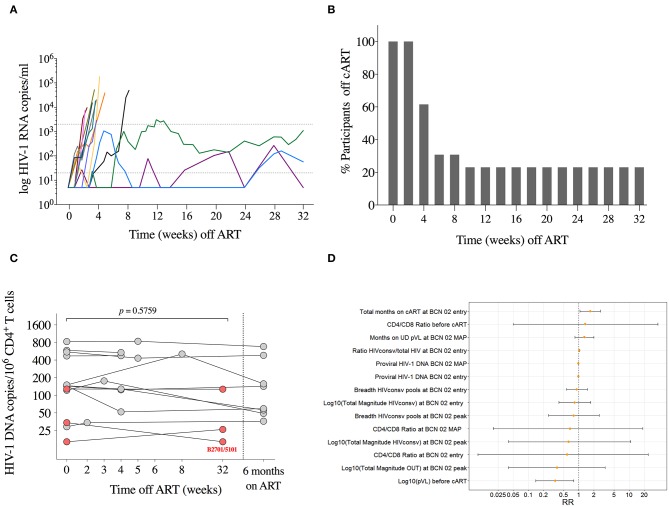
Monitored antiretroviral pause. **(A)** Individual pVL during MAP is shown for each participant in different colors (*n* = 13). Lines are interrupted on day of ART resumption. Dotted lines represent detection limit and threshold for ART resumption (20 and 2,000 copies/ml, respectively). **(B)** Proportion of individuals remaining off ART during MAP. **(C)** Total HIV-1 DNA copies/10^6^ CD4^+^ T cells are shown for each participant at MAP initiation, at day of ART ressumption, and 24 weeks after ART. *P*-value corresponds to comparison between MAP initiation and the day of ART resumption for each individual using the Wilcoxon signed-rank test. Individuals with sustained low-level viremia over 32 weeks are shown in red. **(D)** Estimated relative risks and 95% confidence intervals for the durable control of pVL during MAP obtained from univariate log-binomial regression models.

All participants rebounded (detectable pVL over 20 copies/ml) during MAP (Figure 5A). Median (range) time to first detectable pVL was 13 (7–35) days with median (range) of first detectable pVL of 122 (28–3,410) copies/ml. Ten participants resumed ART before MAP_12_ (MAP-NC for MAP-*Non-controllers*) with median (range) time to resume ART of 28 (16–59) days. All MAP-NC resumed ART due to the viral load criteria, with median (range) pVL of 19,250 (2,900–179,000) copies/ml at the moment of ART resumption (Figure 5B). None of the participants resumed ART due to immune or clinical criteria. A “late-rebounder” presented the first detectable pVL 5 weeks after ART interruption (MAP_5_, pVL of 59 copies/ml) and was able to maintain viral load below 2,000 for 3 more weeks, resuming ART at MAP_8_. In addition to the “late-rebounder,” 3 (23%) other participants remained off ART with sustained pVL <2,000 copies/ml for a total of 32 weeks (MAP-C, from MAP-*Controllers*). Highest peak pVL determination in the MAP-C was of 3,110 copies/ml at week 12 of MAP in the absence of symptoms followed by 2,460 and 1,100 three and six days later (participant decision to stay off). At week 32, two out of the three MAP-C accepted to stay off cART out of the protocol and were followed under standard of care. One MAP-C showed a late rebound after 48 weeks off cART and the other one, voluntary resumed cART after 1.5 years off cART despite sustained low-level viremia. All participants who restarted ART reached viral re-suppression within 6 months. No evidence of emergence of drug resistance was detected.

To assess re-seeding of the viral reservoir during the MAP, total HIV-1 DNA was measured at MAP_0_ (*n* = 13), on the day of ART resumption (*n* = 10), and 6 months after for the 10 MAP-NC (*n* = 8 available), and at MAP_32_ for the three MAP-C. We did not observe any significant change in total HIV-1 DNA during MAP in participants with early ART resumption (Wilcoxon signed-rank, *p* = 0.5759 for MAP-NC) and, noteworthy, nor in the three participants with sustained low level viremia for 32 weeks. Moreover, one of the three MAP-C showed a 2-fold reduction in the HIV-1 DNA (from 34 at MAP_0_ to 16 copies/10^6^ CD4^+^ cells at MAP_32_, Figure 5C). This was the only participant undergoing MAP carrying HLA alleles associated with natural HIV control (HLA-B^*^27:01/HLA-B^*^51:01) (45, 46).

### Factors Influencing Viral Rebound Kinetics

Supplementary Table 1 shows the summary of variables explored to explain the binary outcome defined as MAP-NC vs. MAP-C. The estimated relative risks obtained from the log-binomial models for different covariates analyzed are shown in Figure 5D.

Univariate log-binomial regression models used to detect factors associated with virologic control during MAP revealed that pVL before ART initiation (pre-ART pVL) was the only factor statistically significantly associated with control of viral rebound after ART interruption. For each log increase on the pre-ART pVL, the probability of becoming a MAP-C decreased by 66% (RR 0.34; 95% CI 0.14, 0.79). Interestingly, albeit not statistically significant in the univariate models, the 3 MAP-C had among the lowest reservoir levels at treatment interruption time point (16, 54 and 122 copies/10^6^ CD4^+^ T cells) –consistent with lower pre-ART pVL– and showed the highest shift in CTL immunodominance pattern after vaccination (>700 HIVconsv-specific SFC/10^6^ PBMCs and >75% of HIVconsv dominance at peak immunogenicity time point, Supplementary Figure 6).

## Discussion

In this single-arm, open-label, phase I, proof-of-concept trial performed in HIV-1-infected ART-suppressed individuals treated during acute/recent HIV-1 infection, we show that combination of the HIVconsv vaccines with RMD as a latency reversing agent was safe, highly immunogenic, and induced bursts of viral transcription. The combined intervention resulted in a tendency toward a reduction in proviral DNA levels and was followed by a prolonged viremic control in 23% of participants after ART interruption without evidence of reservoir re-seeding.

Early-treated individuals typically show less immune exhaustion (47) and reduced frequency of immune-escaped viral variants compared to individuals initiating ART at later stages of HIV-1 infection (48), offering a potentially favorable setting to induce a protective immune response upon therapeutic vaccinations. In the parental BCN01 trial, a prime/boost vaccine regimen with ChAdV63.HIVconsv and MVA.HIVconsv induced high frequencies of T cells with high *in vitro* suppressive capacity that markedly shifted the focus of the CD8^+^ CTL response toward HIVconsv sequences that are subdominant during natural infection (20). In the BCN02 trial, after 3 years of viral suppression and 2 years since the last vaccination in BCN01, booster MVA.HIVconsv vaccinations were still immunogenic and further increased breadth, magnitude and immunodominance of CTL responses toward HIVconsv sequences.

Along with a strong vaccine-elicited CTL activity (“kill”), the ability to simultaneously induce reactivation of the viral reservoir (“kick”) is a critical feature for the success of the kick&kill strategy (24). A 3-dose regimen of weekly RMD at 5 mg/m^2^ BSA was selected based on results from previous trials (33, 34). Consistently, we observed a direct *in vivo* effect of RMD in histone 3 acetylation upon each RMD dose, which was followed by changes in cell-associated HIV-1 RNA levels. Conversely, a placebo-controlled dose-escalating trial (ACTG 5315) testing 3 RMD doses (5 mg/m^2^ BSA) administered every two weeks in chronically suppressed individuals, did not show changes in viral transcription (49). The weekly administration regimen and the intensive sampling after each RMD dose in our study allowed us to detect increases in CA HIV-1 RNA above 2-fold in 80% of individuals at any time point after RMD administrations. These changes were followed by consistent increases in T cell activation markers, which remarkably, were maintained one week after RMD_3_, likely reflecting a direct and cumulative effect of weekly RMD dosing and an effective induction of viral transcription. Noteworthy, changes above 2.1-fold in CA-RNA have been estimated to occur in <5% of repeated measurements in an individual (50).

Despite the induction of viral transcription, the kinetics of plasma HIV-1 RNA followed an unclear pattern, similar to previous studies showing variable changes in plasma viremia following LRA administration (34, 51, 52). This variability might reflect suboptimal potency of the agents tested so far and/or the reactivation of predominantly defective proviruses. In the context of the current study, elimination of reactivated cells by vaccine-elicited T cells may have additionally blunted quantification of plasma viremia.

A critical objective of the use of LRA in a kick&kill strategy is to mobilize and ultimately reduce the viral reservoir. Our findings showed that, despite robust immunogenicity of HIVconsv vaccines and at least partial reactivation of the viral reservoir induced by RMD, the net effect on the proviral DNA levels was modest. All participants had detectable levels of HIV-1 DNA at the time of treatment interruption although a tendency toward a decrease by 19.3% from baseline to week 17 (Wilcoxon signed-rank, *p* = 0.0599) was detected. Conversely, a mean 39.7% decrease in reservoir size was observed in the REDUC trial (34). This discrepancy between REDUC and BCN02 results may be explained by the inclusion of early-treated individuals in our study, in which already baseline levels of proviral DNA were substantially lower, challenging the quantification of the effects of the intervention on HIV-1 DNA levels. We acknowledge that the translation of HIV-1 protein expression into antigen presentation—even in case of defective proviruses (1)—upon LRA reactivation is poorly understood. Likewise, the ability of LRA-induced HIV-1 protein expression to effectively induce recognition and killing by CD8^+^ T cells remains to be fully elucidated (53) and therefore, the potential effects of further RMD administrations on the viral reservoir are to be determined. A potential significant toxicity—suggested in *in vitro* assays (54)—on vaccine-induced T cells might also have limited our capability to observe a further reduction in the reservoir size in our study. This hypothesis is consistent with the fact that HIVconsv vaccines induced higher levels of activated T cells compared to Vacc-4x vaccination in the REDUC trial. Nevertheless, the potential toxicity of RMD *in vivo*, its relationship with RMD exposure and, ultimately, whether vaccine-induced T cells were able to sensor and remove infected cells in response to HIV-1 reactivation remains to be determined.

To attain a functional cure, a persistent immune-mediated control of residual HIV-1 might be as relevant as achieving an absolute reduction on the proviral DNA levels. In this regard, the three BCN02 MAP-C, were among the subjects with both lower viral reservoir levels at MAP and higher vaccine-induced responses. In our study, having lower pre-ART pVL was the only outstanding marker associated with viral control during MAP, which correlates with the size of the viral reservoir after ART suppression. These relationships are consistent with previous studies suggesting a role of a low viral reservoir on analytical treatment interruptions (ATI) outcomes (55, 56). Furthermore, after ART discontinuation, MAP-C did not show an initial burst in pVL followed by a fast post-peak control as described in several post treatment controllers (PTC) (8, 57). Collectively, the findings from this and other studies suggest that a small reservoir size, resulting from early ART or another intervention, may be essential to achieve sustained post-intervention control (55) but also, that a potent vaccine-induced immune pressure might contribute to prevent a peak burst of viremia and maintain suppressed viremia for a substantial period of time. This control, mediated by immune pressure, is supported by the absence of re-seeding of the viral reservoir in the BCN02 MAP-C, in contrast to reports from previous ATI trials (58, 59).

The interpretation of the outcome of kick&kill studies may be confounded by individuals controlling HIV rebound after treatment interruption without the need for a prior therapeutic intervention. The prevalence and mechanisms driving such PTC in natural HIV infection are not well-understood. A recent metanalysis (CHAMP study) including 14 interruption trials estimated a 13% rate of PTC among early-treated individuals (57). Importantly, and in contrast to the three BCN02 MAP-C who did not show a transient high burst of viremia, 32% of the 61 PTCs analyzed in the CHAMP metanalysis had peaks of viremia ranging from 1,000 to over 10,000 copies/ml within the first 24 weeks after treatment interruption. Thus, in addition to the different behavior of the 3 MAP-C (23%) with respect to the PTC, according to this metanalysis, the BCN02 trial may have missed additional MAP-C due to the conservative ART resumption criteria used (two consecutive pVL over 2,000 copies/ml).

The safety and tolerability profiles of MVA.HIVconsv and RMD were similar to those reported in previous studies (19, 20, 34). However, there was a SAE in one participant. This case highlights the need for planning intensive monitoring in this kind of pilot trials, even if not powered to detect low-frequency AE, and points toward the need for a trade-off between the number of participants potentially put at risk in well-powered controlled trials and for caution with the use of uncompletely characterized agents in large numbers of individuals. Given that natural PTC rates are considered to be up to 13% in early-treated individuals, powering trials to show viral control efficacy after an ATI becomes challenging (60). Despite frequent clinical monitoring for pVL and access to psychological support, protocol violation during MAP occurred in one individual, probably due to anxiety secondary to the antiretroviral interruption. This case warrants close psychological management in longer term ATIs.

We fully acknowledge the limitations of the small sample size and lack of a control arm in the present study. Therefore, we interpret these results with caution and regard this study only as hypothesis-generating trial for future interventions. BCN02 eligibility was restricted to vaccinated participants in the parental open-label BCN01 trial. This intrinsic restriction limited the sample size to a small number of previously vaccinated individuals and precluded the inclusion of a control arm. At the time of trial design, interventional trials including an ATI were typically small and included very conservative ART resumption criteria (58, 61). Furthermore, ATI acceptability by participants, risks of HIV-1 transmission to others in the absence of available PrEP, and potential viral re-seeding upon treatment interruption were of special concern in early-treated individuals, who had limited viral reservoirs both in size and diversity (48).

Altogether, the results from this pilot study suggest a potential role for kick&kill strategies in inducing durable immune-mediated HIV-1 control in a proportion of early-treated individuals. In view of these results, future controlled studies to identify the mechanisms underlying sustained HIV-1 suppression are warranted.

## Data Availability Statement

All datasets generated for this study are included in the article/Supplementary Material.

## Ethics Statement

The studies involving human participants were reviewed and approved by Hospital Germans Trias i Pujol and Hospital Clínic (AC-15-108-R). The patients/participants provided their written informed consent to participate in this study.

## Author Contributions

BM, JM, BC, and CB conceived and designed the study. CMa, TH, JMM, JM-P, AB, PC, MM, and MV contributed to the study design. CMi, CMa, IR, JT, ACa, MR-U, and MP contributed contributed to data management. BM, MR-U, MP, ML, SM-L, AL, JM, PC, ACa, CMi, SC, AB, and CMa performed the experiments. JM, BM, MP, MV, YA-S, GM, and KL undertook the statistical analysis. ACr and TH contributed with reagents, materials, analysis tools. BM, JM, and CB drafted the manuscript. TH, MR-U, CMa, MP, SM-L, JM-P, JMM, MV, and BC participated in study analyses and revised the manuscript critically for important intellectual content. All authors reviewed and approved the final version of the manuscript.

## Investigators Members of the BCN02

IrsiCaixa AIDS Research Institute-HIVACAT, Hospital Universitari Germans Trias i Pujol, Badalona, Spain: Susana Benet, Christian Brander, Samandhy Cedeño, Bonaventura Clotet, Pep Coll, Anuska Llano, Javier Martinez-Picado, Marta Marszalek, Sara Morón-López, Beatriz Mothe, Roger Paredes, Maria C. Puertas, Miriam Rosás-Umbert, Marta Ruiz-Riol. Fundació Lluita contra la Sida, Infectious Diseases Department, Hospital Universitari Germans Trias i Pujol, Badalona, Spain: Roser Escrig, Silvia Gel, Miriam López, Cristina Miranda, José Moltó, Jose Muñoz, Nuria Perez-Alvarez, Jordi Puig, Boris Revollo, Jessica Toro. Germans Trias i Pujol Research Institute, Badalona, Spain: Ana María Barriocanal, Cristina Perez-Reche. Clinical Pharmacology Unit, Hospital Universitari Germans Trias i Pujol, Badalona, Spain: Magí Farré. Pharmacokinetic/pharmacodynamic modeling and simultation, Institut de Recerca de l'Hospital de la Santa Creu i Sant Pau-IIB Sant Pau, Barcelona, Spain: Marta Valle. Hospital Clinic-HIVACAT, IDIBAPS, University of Barcelona, Barcelona, Spain: Christian Manzardo, Juan Ambrosioni, Irene Ruiz, Cristina Rovira, Carmen Ligero, Emma Fernández, and Jose M. Miró. Projecte dels NOMS-Hispanosida, BCN Checkpoint, Barcelona, Spain: Antonio Carrillo, Michael Meulbroek, Ferran Pujol and Jorge Saz. The Jenner Institute, The Nuffield Department of Medicine, University of Oxford, UK: Nicola Borthwick, Alison Crook, Edmund G. Wee and Tomáš Hanke.

## Conflict of Interest

JMM reports grants and personal fees from Abbvie, Angelini, Contrafect, Genentech, Gilead, Jansen, Medtronic, MSD, Pfizer, ViiV Healthcare, outside the submitted work. TH reports grants from Medical Research Council UK, during the conduct of the study, and has a patent US 7981430B2 issued. CB was founder, CSO and shareholder of AELIX THERAPEUTICS, S.L. BM was a consultant for AELIX THERAPEUTICS, S.L., outside the submitted work. The remaining authors declare that the research was conducted in the absence of any commercial or financial relationships that could be construed as a potential conflict of interest.
