# Supplementary material for: HIVconsv Vaccines and Romidepsin in Early-Treated HIV-1-Infected Individuals: Safety, Immunogenicity and Effect on the Viral Reservoir (Study BCN02)
Source: Front Immunol. 2020 May 6;11:823. doi: 10.3389/fimmu.2020.00823 (PMC7218169; doi:10.3389/fimmu.2020.00823)
Supplement: Supplementary file 1 [file Data_Sheet_1.docx]

# Supplementary materials

**
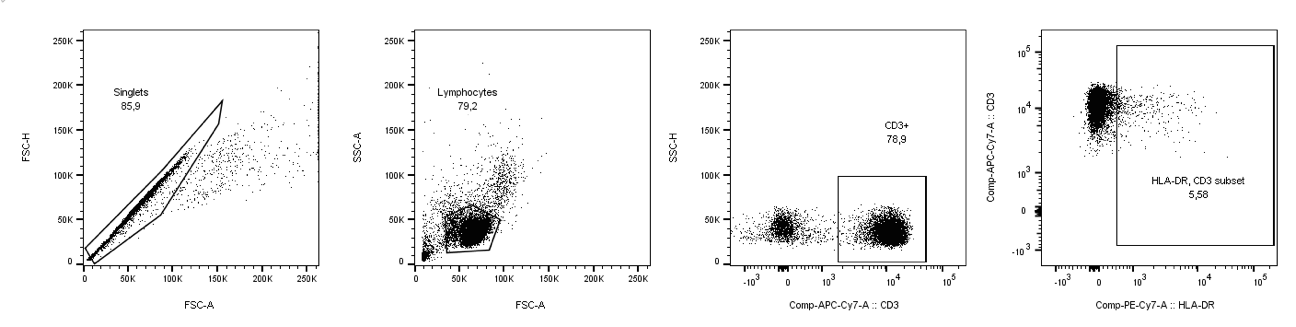
**

**Figure 1.** Gating strategy for T-cell activation analysis based on HLA-DR expression in CD3 T cells: Singlet gate (FSC-H vs. FSC-A) -> Lymphocyte gate (SSC-A vs. FSC-A) -> CD3+ T cells (SSC-H vs. CD3) -> CD3+ HLA-DR+ (CD3 HLA-DR).

**Figure 2**. Longitudinal CD4+ T cells counts during the 3 RMD infusions is shown for all 15 participants. Median values at each timepoint is shown with a red dot. P-values correspond to comparisons between the indicated time points using the Wilcoxon signed-rank test.

**
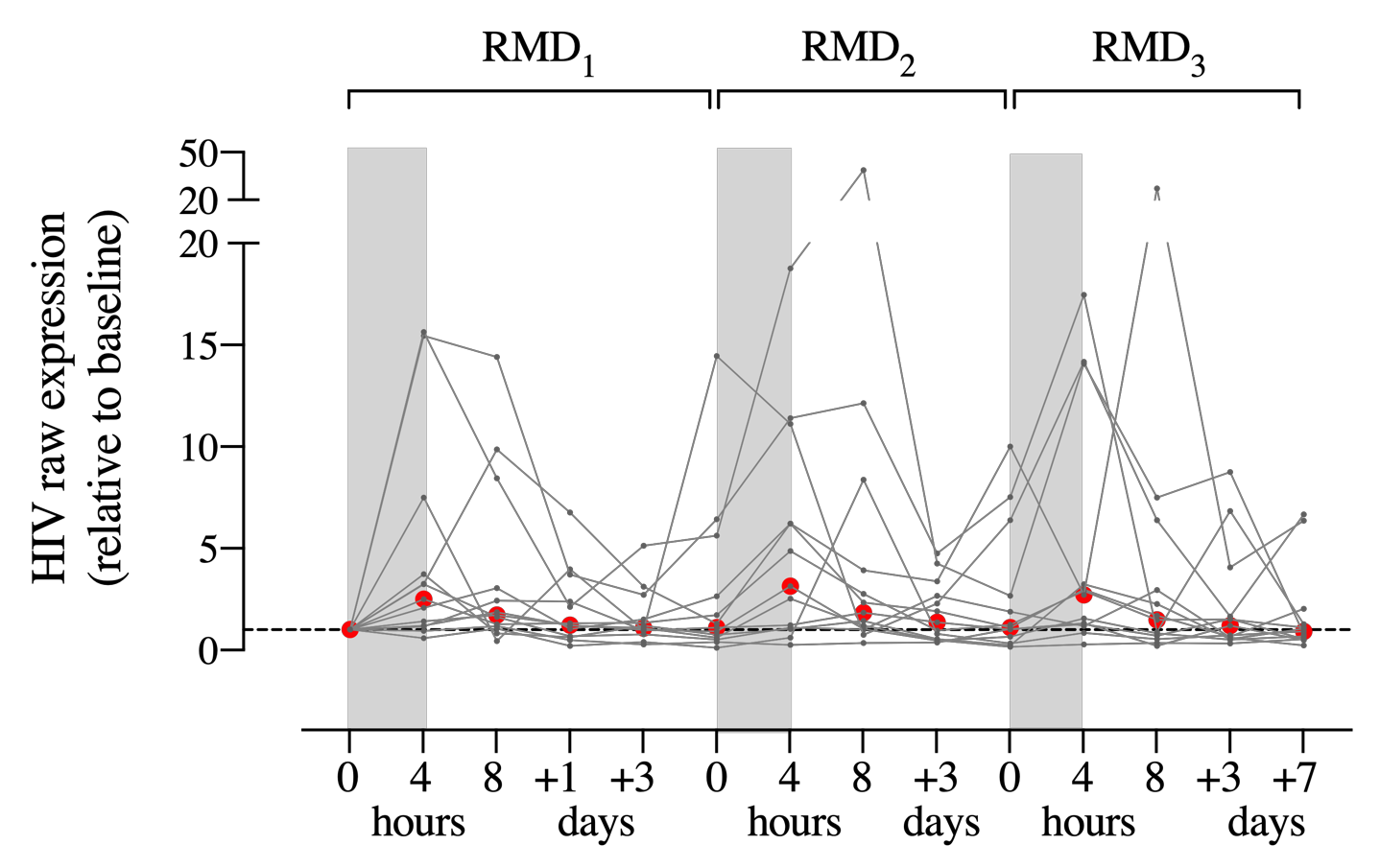
**

**Figure 3.** Changes in individual cell-associated HIV-1 RNA measurements in peripheral CD4+ T-cells. Data is shown without correction by housekeeping gene expression levels and relative to baseline expression levels, i.e. before RMD1. Median values at each timepoint is shown with a red dot. At the end of each RMD infusion, a median increase of the viral expression is detected higher than 2.5-fold, with normalization beyond 4hours.


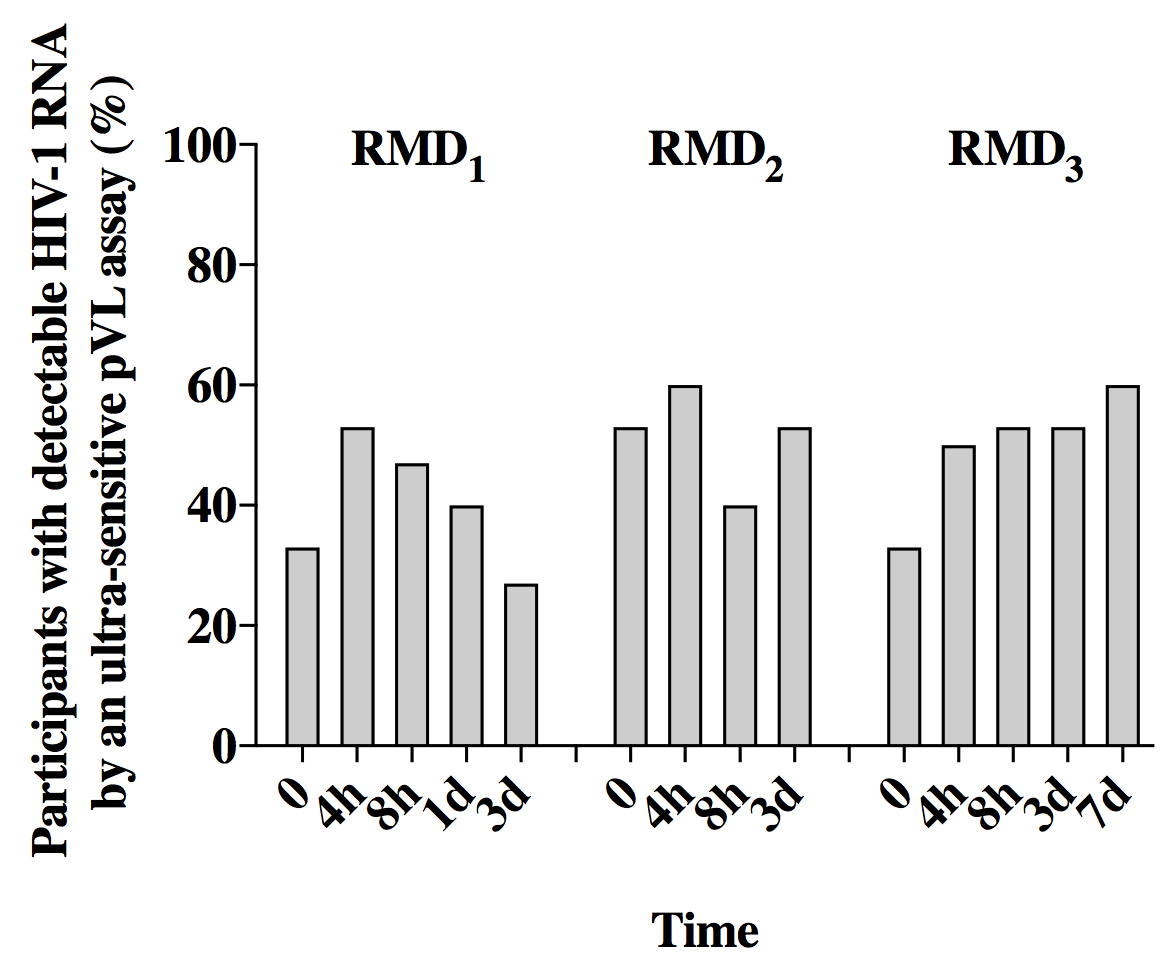


**Figure 4.** Proportion of participants with detectable HIV-1 RNA during the three RMD administrations by an ultra-sensitive viral load assay using 4-8 ml plasma.

**

Figure 5.** Responses to individual HIVconsv peptide pools (P1-P6) for each participant (n=15). The response breadth is calculated as the number of peptide pools giving a positive response during the trial (out of 6 pools) in the ex-vivo ELISPOT assay and the number of individual HIVconsv OLP giving a positive response in the ELISPOT using in-vitro expanded PBMC at peak immunogenicity time point is indicated.

**
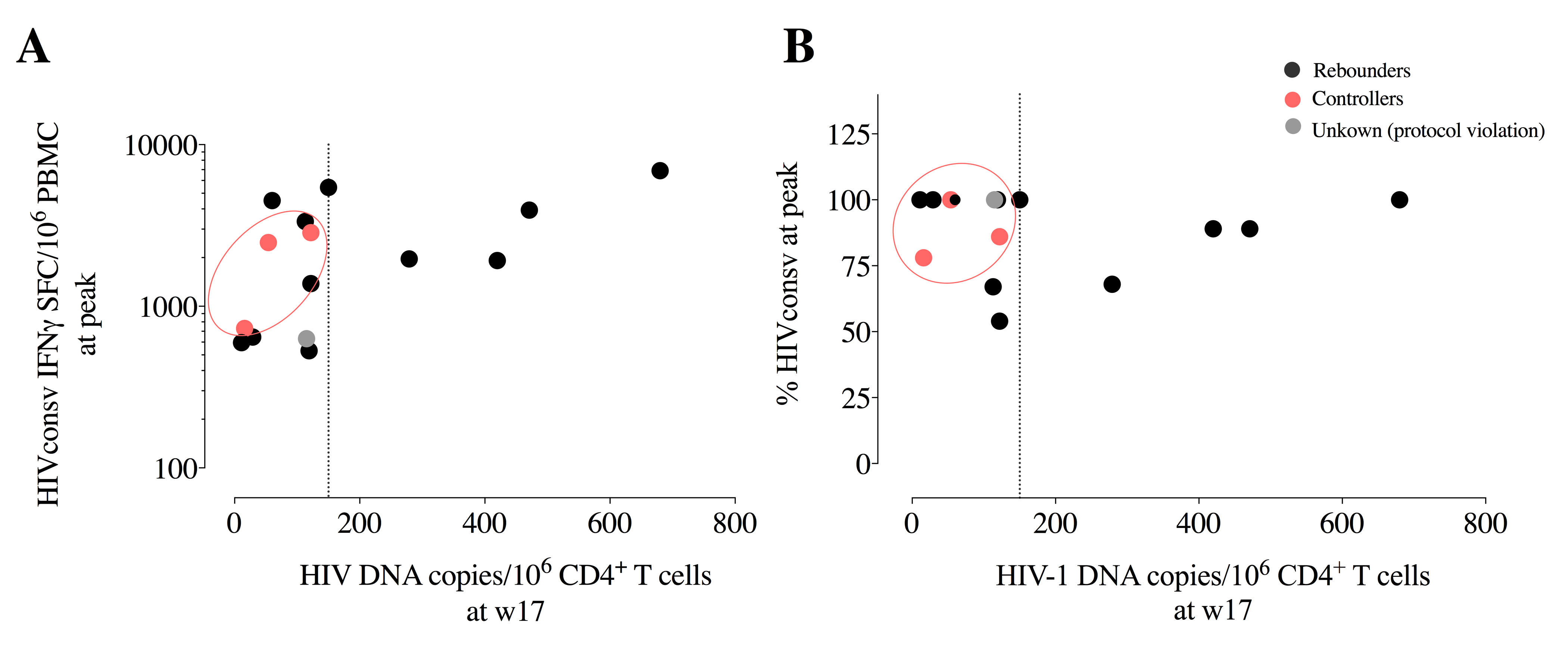
**

**Figure 6**. Distribution of proviral DNA levels after RMD and vaccine elicited responses at peak immunogenicity time point among the 15 participants.

**Table 1**. **Summary of continuous covariates**

| **Table S1.** Summary of continuous variables included in the log-binomial models | | | | | | | | | |
| --- | --- | --- | --- | --- | --- | --- | --- | --- | --- |
|  | **MAP - *Non Controllers* (n=10)** | | | |  | **MAP - *Controllers* (n=3)** | | | |
| **Variable** | **Med** | **IQR** | **Min** | **Max** |  | **Med** | **IQR** | **Min** | **Max** |
| **Demographics** | | | | | | | | | |
| Age | 42 | 38-45 | 33 | 48 |  | 32 | 31-36 | 30 | 40 |
| **Before any ART initiation** | | | | | | | | | |
| Days from HIV-1 to ART | 82 | 67-107 | 32 | 118 |  | 112 | 70-138 | 28 | 164 |
| log_10_ (pVL) before any ART | 5.02 | 4.88-5.16 | 4.26 | 5.48 |  | 3.35 | 3.28-4.59 | 3.20 | 5.82 |
| CD4 absolute before any ART | 453 | 382-560 | 299 | 785 |  | 631 | 608.5-633 | 586 | 635 |
| CD4/CD8 ratio before any ART | 0.56 | 0.45-0.69 | 0.44 | 1.26 |  | 0.56 | 0.42-0.85 | 0.27 | 1.14 |
| **At BCN02 entry** | | | | | | | | | |
| CD4 absolute | 728 | 533-1331 | 416 | 1408 |  | 657 | 652.5-814 | 648 | 971 |
| CD4/CD8 ratio | 1.37 | 1.22-1.47 | 1.00 | 1.93 |  | 1.33 | 1.15-1.54 | 0.97 | 1.74 |
| Total months on ART | 38.88 | 37.56-40.20 | 36.36 | 41.04 |  | 41.64 | 39.30-42 | 36.96 | 42.36 |
| Months on UD pVL | 35.52 | 35.40-35.88 | 31.32 | 40.32 |  | 36.36 | 36.18-39 | 36 | 41.64 |
| **At MAP** | | | | | | | | | |
| CD4 absolute | 735 | 484-1075 | 468 | 1269 |  | 854 | 675-902 | 496 | 950 |
| CD4/CD8 ratio | 1.30 | 1.25-1.48 | 0.87 | 1.75 |  | 1.52 | 1.14-1.57 | 0.76 | 1.61 |
| Total months on ART | 41.88 | 44.04-46.32 | 41.88 | 46.80 |  | 46.80 | 44.88-47.28 | 42.96 | 47.76 |
| Months on UD pVL | 41.52 | 40.92-43.68 | 37.80 | 45.60 |  | 42.48 | 42.24-44.34 | 42 | 46.20 |
| **Vaccine immunogenicity** | | | | | | | | | |
| HIVconsv magnitude |  |  |  |  |  |  |  |  |  |
| At BCN02 entry | 160 | 0-287 | 0 | 2640 |  | 0 | 0-655 | 0 | 1310 |
| At BCN02 peak | 1965 | 1380-3940 | 530 | 6901 |  | 2480 | 1605-2668 | 730 | 2855 |
| HIVconsv pools breadth |  |  |  |  |  |  |  |  |  |
| At BCN02 entry | 1 | 0-2 | 0 | 2 |  | 0 | 0-1 | 0 | 2 |
| At BCN02 peak | 4 | 4-6 | 3 | 6 |  | 6 | 5.5-6 | 5 | 6 |
| HIVconsv immunodominance |  |  |  |  |  |  |  |  |  |
| At BCN02 entry | 6 | 0-8 | 0 | 37 |  | 0 | 0-38 | 0 | 76 |
| At BCN02 peak | 89 | 68-100 | 54 | 100 |  | 86 | 82-93 | 78 | 100 |
| Responses OUTside HIVconsv |  |  |  |  |  |  |  |  |  |
| At BCN02 entry | 4525 | 3088-5385 | 1635 | 8945 |  | 3328 | 1872-3428 | 415 | 3528 |
| At BCN02 peak | 705 | 405-1150 | 130 | 5385 |  | 530 | 325-925 | 120 | 1320 |
| **Viral reservoir** | | | | | | | | | |
| Week 0 BCN02 | 190 | 107-434 | 18 | 752 |  | 65 | 62.5-116.5 | 60 | 168 |
| Week 3 BCN02 | 157 | 110-494 | 26 | 892 |  | 46 | 34.5-100 | 23 | 154 |
| Week 6 BCN02 | 131 | 105-464 | 60 | 656 |  | 43 | 36.5-185.5 | 30 | 328 |
| Week 17 BCN02 | 144 | 119-420 | 29 | 680 |  | 54 | 35-88 | 16 | 122 |
| **RMD-PK** | | | | | | | | | |
| AUC_1_ | 392.9 | 386-427.1 | 314 | 439.5 |  | 473.5 | 432.5-548.9 | 391.5 | 624.3 |
| ART: antiretroviral treatment; UD pVL: undetectable plasma viral load; MAP: Monitored antiretroviral pause; RMD: Romidepsin | | | | | | | | | |
